# IL-17 Expression in Dermatitis Herpetiformis and Bullous Pemphigoid

**DOI:** 10.1155/2013/967987

**Published:** 2013-07-21

**Authors:** Agnieszka Zebrowska, Malgorzata Wagrowska-Danilewicz, Marian Danilewicz, Olga Stasikowska-Kanicka, Anna Cynkier, Anna Sysa-Jedrzejowska, Elzbieta Waszczykowska

**Affiliations:** ^1^Department of Dermatology and Venereology, Medical University of Lodz, 5 Krzemieniecka Street, 94-017 Lodz, Poland; ^2^Laboratory of Nephropathology of Medical University of Lodz, 251 Pomorska Street, 92-213 Lodz, Poland

## Abstract

Dermatitis herpetiformis (DH) and bullous pemphigoid (BP) are skin diseases associated with eosinophilic and neutrophilic infiltrations. Although cytokines are critical for the inflammatory process, there are single findings concerning concentration of IL-17 in bullous diseases. The goal of this study was to assess IL-17 expression in DH and BP patients. Skin biopsies were taken from 10 DH, 14 BP patients and from 10 healthy subjects. The localization and expression of IL-17 was studied by immunohistochemistry and the serum concentration was measured by immunoassays. Expression of IL-17 in the epidermis and in influxed cells in dermis was detected in skin biopsies. Expression of IL-17 was statistically higher in epidermis and infiltration cells in specimens from BP than from DH patients. Examined interleukin expression was detected in perilesional skin of all patients but it was much lower than in lesional skin. The expression of IL-17 was not observed in biopsies from healthy people. Serum level of IL-17 was statistically higher in BP and DH groups as compared to control group. Our results provide the evidence that IL-17 may play an essential role in activating and recruiting eosinophils and neutrophils, which ultimately contribute to the tissue damage in DH and BP.

## 1. Introduction

Dermatitis herpetiformis (DH) is one of the subepidermal autoimmune bullous diseases characterized by skin and intestinal lesions. Skin lesions include polymorphic eruption accompanied by severe pruritus. Intestinal lesions are characterized by atrophy of intestinal villi resulting from immunological process [1]. Diagnosis of DH is established on the results of direct immunofluorescence test (DIF) revealing granular deposits of IgA in the papillae and the presence of circulating IgA antibodies directed against endomysium and/or tissue and epidermal transglutaminase (tTG, eTG) [2, 3]. Skin lesions in DH are histologically characterized by neutrophilic infiltrate leading to destruction of basement membrane zone (BMZ) proteins, anchoring fibers, and blister formation [4–6].

Bullous pemphigoid (BP) is a blistering disease, characterized by inflammatory infiltrate in the dermis, presence of IgG and C3 deposits along the basement membrane zone, and circulating IgG autoantibodies. Autoantibodies binding to autoantigens (glycoproteins: 230 kD (BPAG1) and 180 kD (BPAG2)) localized in the basement membrane of the epidermis activate a series of immunological and enzymatic phenomena leading to destruction of basement membrane components and anchoring fibers and blister formation as in DH [7, 8].

Inflammatory infiltrates in the dermis, formed by eosinophils and neutrophils and bound *in vivo* deposits along the basement membrane in BP or in the top of papillae in DH, are observed. Ultrastructural studies also confirmed the presence of intensive inflammatory infiltrate at dermo-epidermal junction, as well as destruction of hemidesmosomes and components of extracellular matrix [9].

Formation of the infiltrates is preceded by early accumulation of leukocytes, depending on activity of adhesion molecules. The binding of autoantibodies leads to activation of keratinocytes, release IL-6 and IL-8, of activation of C5 component of the complement and metalloproteinases—enzymes produced by eosinophils and neutrophils attracted to the basement membrane by selectins and integrins and chemokines [10, 11]. Chemokines are important chemoattractants for both eosinophils and neutrophils [12, 13]. Chemokines and cytokines play their role through receptors. Some of them are highly specific whereas others may interact with more than one ligand [12, 13].

Few studies available suggested cytokines' role in generation of inflammatory influx in autoimmune blistering diseases [14–17].

There is increasing evidence that Th17 cells and the cytokines they release such as interleukin-17 (IL-17) are important regulators of innate and adaptive immune responses in many Th1- and/or Th2-mediated autoimmune diseases such as rheumatoid arthritis, systemic lupus erythematosus, and allergic asthma [18]. There is also evidence that Th17 cells may have a role in pathogenesis of blistering skin diseases. Interleukin-17 is important in initiation and maintenance of many autoimmune reactions and it is involved in production of proinflammatory cytokines, matrix metalloproteinases, neutrophils, and eosinophils, all of which are important pathogenic factors in bullous pemphigoid and dermatitis herpetiformis [19]. The hypothesis is that interleukin-17 may have an important pathogenic role in DH and BP.

It was previously reported that Th17 cells are recruited to the lesional skin in pemphigus vulgaris (PV) and pemphigus foliaceus (PF). Immunohistochemical studies showed that both IL-17+ and Foxp3+ cells were present in higher numbers in cells in BP lesions, compared with control skin. IL-17/CD4 ratio in BP was significantly higher than that in PF. Foxp3/CD4 ratio in BP was significantly lower than that in either PV or PF. There were no obvious correlations between these cells and the disease severity of BP. The previous study suggests that, compared with pemphigus, BP shows more Th17 cell-related inflammation and less Treg-related regulation [20]. There was no data about IL-17 serum levels in blistering disease comparing this level with tissue expression. There was no literature data with reference to the role of IL-17 in pathogenesis of dermatitis herpetiformis.

Il-17 can be assessed in the skin lesions and sera of patients and can be used as a marker of disease activity and response to therapy. The information obtained could also lead to the development of novel therapeutic strategies for this and other autoimmune blistering diseases.

The goal of this study was to assess interleukin-17 expression in skin lesions and perilesional area and serum levels in patients with DH and BP.

## 2. Materials and Methods

### 2.1. Patients

The study included 34 persons: 14 untreated patients with BP (range: 58 to 84 years, average: 68,5) and 10 with DH (range: 18 to 70 years, average: 49,8) in an active stage of the disease. A control group consisted of 10 healthy individuals (range: 19 to 80 years, average: 52,6 years).

All the patients signed informed consent before entering the study and the study protocol (RNN/132/07/KB) was approved by The Local Ethical Committee of Medical University of Lodz.

Eight out of 10 DH patients had skin lesions characterized by vesicles and itching papules; the others had erythematous papules. In all the cases histological pictures showed perivascular neutrophilic infiltrates, the presence of Pierrard's abscesses, and in all patients small subepidermal blisters. In 7/10 samples large unilocular blisters displaying multiple neutrophilic papillary microabscesses were found. The histopathologic findings according to Ackerman in all cases were fully developed [21]. Direct immunofluorescence tests revealed the presence of granular deposits of IgA in skin papillae and indirect immunofluorescence tests were positive for IgAEmA (Oesophagus Monkey IgAEmA, Medizinische Labordiagnostica) in all the patients (titer 1 : 40–1 : 640, median 1 : 80). Antitissue transglutaminase antibodies measured using an immunoassay (Celikey, Pharmacia & Upjohn) were present in 7/10 cases (median 5.1 IU/mL (range: 0,0–186,3; IU/mL). Diagnosis of DH was established based on clinical presentation and results of histological and immunological examination.

Pemphigoid was diagnosed based on clinical picture and histological and immunological findings [7]. The patients were at an active stage of the disease. In 12 out of 14 patients skin blisters, vesicles, and itching papules were found, whereas others had only small vesicles and urticarial papules. The histopathologic findings according to Ackerman in all cases were fully developed [21]. In all the patients direct immunofluorescence test revealed IgG/C3 linear deposits along BMZ. In salt split test deposits were observed in epidermal part of the blister. Using indirect immunofluorescence test circulating IgG antibodies were found in 14/14 patients, whereas ELISA test showed the anti-NC16 autoantibodies (MBL, Nagoya, Japan) present in serum of 11 out of 14 patients. Typical histological features of BP including neutrophilic infiltrates, eosinophils, lymphocytes, and, in 12 cases subepidermal blisters supported the clinical diagnosis.

#### 2.1.1. Tissue Specimens

 The biopsies were taken from the buttock or trunk skin before administration of any (topical or systemic) treatment. Skin lesions lasted between 2 weeks and 3 months. Biopsy specimens were taken from buttock skin of healthy volunteers. 

#### 2.1.2. Immunohistochemistry

Paraffin-embedded sections (3-4 *μ*m thick) were used for routine H&E staining and for immunohistochemistry with DAKO EnVision detection system using immunoperoxidase method. The following primary monoclonal antibodies were used: antiinterleukin 17 obtained from R&D, UK.

 For immunohistochemistry the paraffin-embedded sections were placed on adhesive plates, dried at 56°C for 24 hours, and later deparaffinized in a series of xylenes and alcohols with decreasing concentrations. Activity of endogenous peroxidase was inhibited with 3% hydrogen peroxide solution in methanol for 5 minutes.

 In order to retrieve the antigenicity of tissues and allow them to react with antibodies, specific procedures were used for each of the tested antibody, according to manufacturers' instruction. After incubation with diluted antibodies for 60 minutes at room temperature, they were washed with Tris buffer twice. DAKO EnVision double-step visualization system was then applied in order to visualize the antigen-antibody reaction. In cases of positive immunohistochemical reaction cellular nuclei were stained with the Meyer haematoxylin for 2 minutes. After dehydration and processing through series of acetones and xylenes the sections were fixed in Canadian balm.

#### 2.1.3. Morphometry

Histological morphometry was performed by means of image analysis system consisting of a PC equipped with a Pentagram graphical tablet, Indeo Fast card (frame grabber, true color, real time), produced by Indeo (Taiwan), and color TV camera Panasonic (Japan) coupled to a Carl Zeiss microscope (Germany). This system was programmed (MultiScan 8.08 software, produced by Computer Scanning Systems, Poland) to calculate the number of objects (semiautomatic function). The coloured microscopic images were saved serially in the memory of a computer, and then quantitative examinations had been carried out. The IL-17-positive cells were counted (semiautomatic function) in a sequence of 7–10 consecutive computer images of 400× high power fields—0.0047 mm^2^ each. The results were expressed as percentages of IL-17-positive cells of all lymphocytes determined by their morphology. In each case 500 lymphocytes were counted. 

### 2.2. Statistical Methods

All values were expressed as the mean ± SD (standard deviation). Differences between groups were tested using unpaired Student's *t*-test preceded by evaluation of normality and Levene's test. The Mann-Whitney *U* test was used where appropriate. Results were considered statistically significant if *P* < 0.05. 

### 2.3. Serum IL-17 Levels

IL-17 levels were measured in serum in all patients and healthy controls undergoing skin biopsy. Five cc of venous blood were drawn from the ulnar vein and after centrifugation serum was stored at −20°C for an immunoassay.

The enzyme-linked immunoassays were used to measure IL-17. Immunoassays were obtained from R&D, UK.

IL-17 levels are shown in pg/mL as mean +/− SD whereas t-TN are presented in IU/mL. The Mann-Whitney test was applied in statistical analysis and results with *P* < 0.05 were considered statistically significant.

## 3. Results

**Figure 1:**
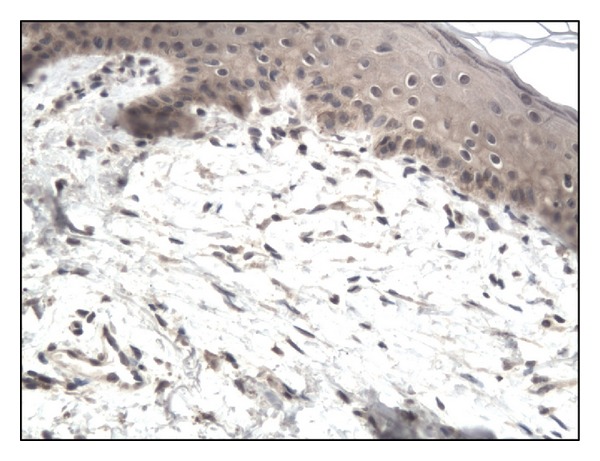
Almost negative immunoexpression of IL-17 in normal skin. Mag. 200x.

**Figure 2:**
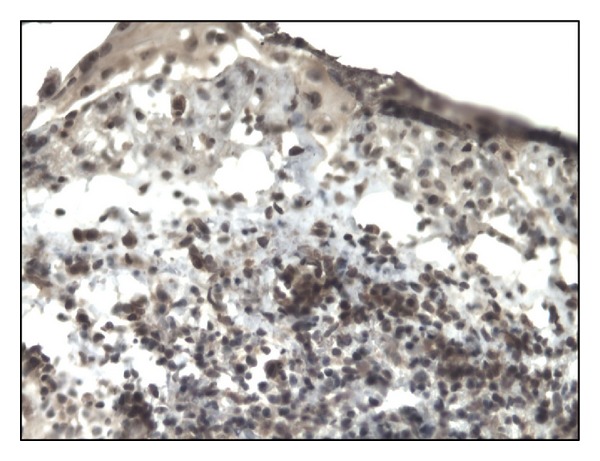
Intense immunoexpression of IL-17 on numerous lymphocytes in BP patient (lesion). Mag. 200x.

**Figure 3:**
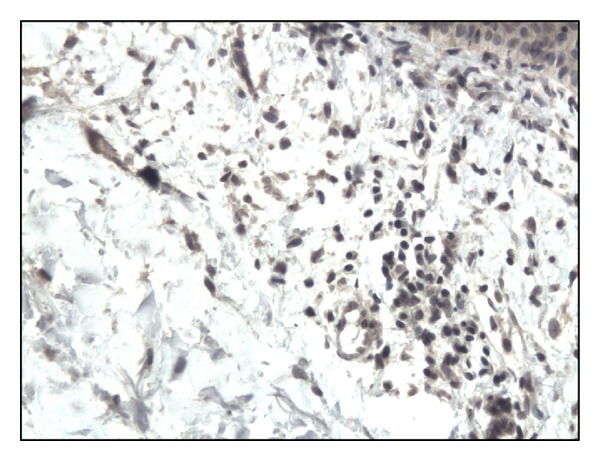
Moderate immunoexpression of IL-17 on lymphocytes in BP patient (surrounding tissue) Mag. 200x.

**Figure 4:**
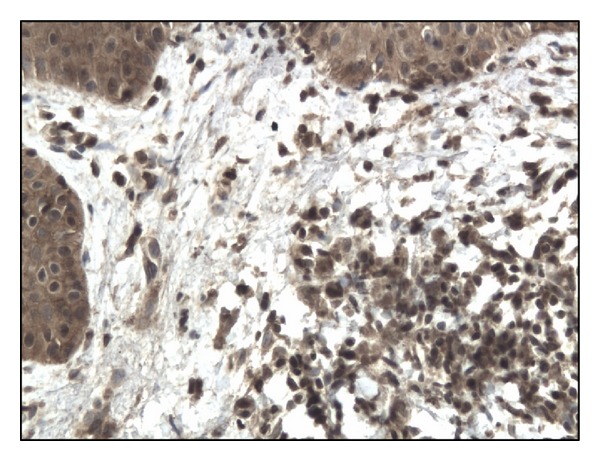
The immunoexpression of IL-17 on lymphocytes in DH patient (lesion). Mag. 200x.

**Figure 5:**
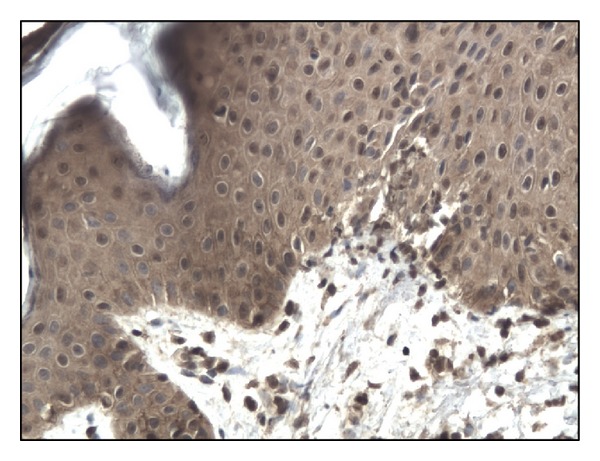
Moderate immunoexpression of IL-17 on lymphocytes in DH patient (surrounding tissue). Mag. 200x.

In controls staining for IL-17 was almost negative (Figure 1). In both bullous pemphigoid and dermatitis herpetiformis groups IL-17-positive cells (lymphocytes) were detected in the upper dermis of the skin along the basement membrane and around vessels (Figures 2, 3, 4, and 5). 

The quantitative data of IL-17-positive cells expressed as percentages of all lymphocytes in BP and DH appear from Table 1.

In biopsies of perilesional skin Il-17 expression was present in all patients with BP. In majority of patients moderate intensity of Il-17 expression was present in skin lesions, whereas in six patients strong IL-17 expression was observed. Expression in lesional skin and perilesional skin is statistically higher in skin lesions (in BP *P* < 0.003 and in DH *P* < 0.05).

Similar data were obtained from lesions biopsies in patients with DH. Although Il-17 expression was present in all patients, in one patient the expression was low. In skin biopsies of perilesional area and skin lesions taken from patients with DH, Il-17 expression was weaker than in patients with BP (1.9 ± 1.2 versus 2.3 ± 2.1 and 3.9 ± 2.8 versus 7.4 ± 4.1, *P* < 0.001, resp.).

### 3.1. Serum IL-17 Levels

**Figure 6:**
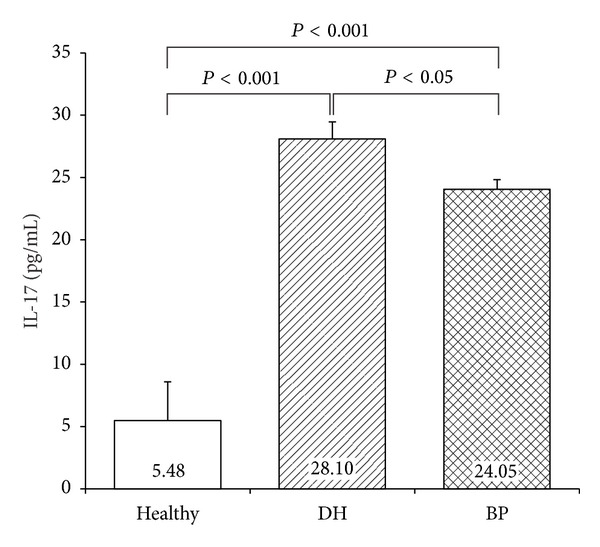
Serum Il-17 concentration in DH, BP, and control groups.

IL-17 levels were statistically higher in DH patients (28.10 +/− 1.36) as compared to patients with BP (24.05 +/− 0.78, resp., *P* < 0.05) (Figure 6). IL-17 concentrations in DH and BP patients compared to healthy subjects were statistically higher (*P* < 0.001) (Table 2).

## 4. Discussion

 Pathogenesis of bullous diseases is not known, although several aspects of their pathogenesis have been already elucidated [1, 3, 7, 10]. BP and DH are diseases characterized by inflammatory changes in area surrounding skin lesions typical for the disease including (but not limited to) an increase in vessel and connective tissue adhesion molecules expression, an influx of various inflammatory cells, local as well as systemic increase in proinflammatory cytokine synthesis. Due to differences in composition of an inflammatory cells in both DH and BP, it was of interest to analyze the IL-17 expression in skin lesions, which might play an important role in selective recruitment of leukocytes to the skin.

In this paper we were investigating the expression of Il-17 in skin lesions and perilesional skin and blood concentration in well-described patients with BP and DH. We compared patients' results with healthy subjects'. 

The pathogenesis of inflammatory skin disease involves the release of cytokines from keratinocytes, and one of these, IL-1*β*, has been previously implicated in inflammatory skin disease. Th17 cells, a subset of Th cells involved in autoimmunity and inflammation, possess IL-1*β* receptors and secrete cytokines such as IL-17 and IL-22 in response to IL-1*β* stimulation. A mutation in the inflammasome protein NLRP3 (NACHT, LRR, and PYD domains containing protein 3) causes excess production of IL-1*β*, resulting in an augmentation of Th17-dominant pathology. These results indicate that cytokines from Th17 cells may potentiate IL-1*β*-mediated skin inflammation and result in phenotypic alterations of keratinocytes via a feedback mechanism [22]. 

Tumor necrosis factor-(TNF-) *α* is known to play a pivotal role in the pathogenesis of psoriasis. TNF-*α* has been shown to act directly on keratinocytes, thereby inducing the production of various kinds of chemokines, which contributes to the infiltration of leucocytes into the psoriatic lesions. Recent studies have shown that both interleukin IL-17 and IL-27 are increased in psoriatic lesional tissue. However, the interactions between TNF-*α*, IL-17 and IL-27 in chemokine production by keratinocytes have not been fully elucidated. Fujiwara et al. found that IL-17, and IL-27 exert opposite effects on TNF-*α*-mediated chemokine production. This suggests that lesional balance of IL-17 and IL-27 is involved in the recruitment of T cells, natural killer cells, neutrophils, monocytes, or dendritic cells, thereby affecting inflammation in skin diseases [23].

In our recent paper we have showed a high expression of TNF-*α* in skin lesions in BP and DH. It has been also postulated that the process of blister development might be driven by many factors including local metalloproteinases overexpression, mediated by cytokines. Various proinflammatory cytokines, that is, TNF, are involved in many biological processes, among others is overproduction of metalloproteinases, especially stromelysins, gelatinases, and matrilysins.17 [24].

Th17 cells, characterized by interleukin-17 production, play crucial roles in the pathogenesis of autoimmune diseases [25]. A potential role of Th17 cells in BP was recently suggested, because an increased recruitment of IL-17+ cells in the lesional tissue was observed in mucous membrane pemphigoid (MMP), another pemphigoid member [26]. Although the role of Th17 cells in the pathogenesis of autoimmune diseases is unresolved, they may be the initiators of diseases [20]. Alternatively, Th17 cells may possibly appear in a protective response to maintain epithelial homoeostasis [27]. In fact, IL-17 production was induced by keratinocytes in an *in vitro* system [28]. If the latter is the case, the more severe disruption in epithelial integrity in BP and PV, when compared with PF, may increase the number of regional Th17 cells.

The second intriguing issue is that the number of Foxp3+ cells in BP was significantly smaller than that in pemphigus groups. In fact, a decreased number or impaired functions of circulating Treg cells in several autoimmune diseases have been reported [20].

There unfortunately is still vast amount of data supporting the role of Il-17 in pathogenesis of autoimmune bullous diseases. According to our knowledge, no published data are available about IL-17 tissue expression in DH. In our hands IL-17 expression was higher in patients with BP as compared to patients with DH. These findings are in contrast with data concerning Il-17 concentration in sera in patients. In DH patients IL-17 serum level was higher in comparison to patients suffering from BP, although this difference in serum concentration between DH and BP patients was smaller than between DH and BP and control group. 

Inflammatory changes in skin in both studied diseases are related not only to neutrophilic influx but also to eosinophils aggregation within area of involved skin [21]. Several papers discussed the role of various factors in eosinophils infiltration. Our data may suggest that different CCR1 expression may take part in eosinophils infiltration in BP [29]. 

Eosinophils are provided with a preformed or newly generated mediators. Th2 cells are involved in all stages of immune response in the course of the allergic reaction [30, 31], and studies carried out so far indicate that these cells are also the source of many proinflammatory cytokines and chemokines involved in allergic inflammation: IL-4, -5, -10, and -13 [32, 33]. It has been shown that expression of mRNA for cytokines associated with lymphocytes Th2 (especially IL-5) and the concentration of these cytokines in the tissues of an allergic reaction correlate with the number of excited eosinophil's purposes [34, 35] which indicates that the accumulation and activation of lymphocytes CD4+ T cells are directly related to the induction of eosinophilic infiltration. 

Among the recently discovered cytokines (1993) proinflammatory synthesized by activated T lymphocytes, CD45RO + memory, both the auxiliary (CD4+) and cytotoxic (CD8+), is a group belonging to the family of cytokines interleukin 17 (IL-17) [36, 37]. There is numerous scientific evidence that these cytokines play an important role in acute and chronic inflammations [36, 37], including also the pathogenesis of allergic diseases [38]. It is believed that IL-17A (which is a prototype cytokine that group) can—in the mechanism similar to that described above, the relationship between activity and the development of Th2 cells infiltration, development of eosinophilic influx,—an important link to in an indirect way it combines synthesizing cells T with an increased influx to the site of reaction inflammatory neutrophil [37, 39]. Numerous studies have shown that increased IL-17A activity leads to local flow neutrophils for respiratory purposes, mainly through the influence of this cytokine to synthesize stromal cells chemotactic factors for neutrophils purposes (such as CXC chemokines) and growth factors for these cells (G-CSF, GM-CSF) [36, 40]. 

During activation of eosinophils, these cells start secrete LTC4, lipoxins, PAF, TXA2 and PGE2. In addition, proteins secreted by eosinophiles such as IL-13, Il-5, GM-CSF, eotaxin and RANTES activate an autocrine mechanism of the same cell [41]. Synthesis of eosinophilic cells, IL-1, IL-4, and IL-16 suggests that they may affect the maturation and activation of other cells [40]. Similarly, in our study, it is confirmed that these cells are activated in the process of formation changes primarily in patients with BP, where the concentrations of eotaxin and other chemokines were significantly higher than in patients with DH and healthy subjects [29]. Extremely important is the impact of eosinophil activity markers on other factors such as the cytokine network of adhesion molecules and tissue factor.

The efficacy and safety of neutralizing IL-17 itself are under clinical investigation in autoimmune settings, but initial reports suggest different responses in psoriasis and rheumatoid arthritis [42]. Clearly more work is required to fully understand the relevance of the IL-23/IL-17 connection in the spectrum of autoimmune disease. 

Action of autoantibodies in BP and DH cannot fully explain several important features of the diseases such as the difficulty of transferring with the pathogenic autoantibodies or the presence of heavy lesional infiltration of eosinophils and neutrophils which is necessary for disease production, although it has a lot of immune mechanisms and relations between them in bullous diseases described [43]. The hypothesis is that interleukin-17 has an important pathogenic role in BP and DH and can describe features of the disease not explained by the autoantibody theory. This cytokine can be assessed in the sera of patients and can be used as a marker of disease activity and response to therapy. The information obtained could also lead to the development of novel therapeutic strategies for this and other autoimmune blistering diseases.

## 5. Conclusions

 BP and DH are diseases with unknown pathogenesis, varying in clinical picture, and have many common issues regarding the inflammatory changes in skin lesions.

Inflammatory changes in skin in both studied diseases are related not only to neutrophilic influx but also to eosinophils aggregation within area of involved skin [18]. Several papers discussed the role of various factors in eosinophils infiltration.

There is vast amount of data supporting the role of Il-17 in pathogenesis of autoimmune bullous diseases. In our hands IL-17 expression was higher in patients with BP as compared to patients with DH. These findings are in contrast with data concerning Il-17 concentration in sera in patients. In DH patients IL-17 serum level was higher in comparison to patients suffering from BP, although this difference in serum concentration between DH and BP patients was smaller than that between DH and BP and control groups. 

IL-17 can be be used as a marker of disease activity and response to therapy.

## Figures and Tables

**Table 1:** The quantitative data of IL-17-positive cells expressed as percentages of all lymphocytes in bullous pemphigoid (BP) and dermatitis herpetiformis (DH).

Cases	BP (%)	DH (%)	*P* value (BP versus DH)
Skin lesions	7.4 ± 4.1	3.9 ± 2.8	*P* < 0.001
Perilesional skin	2.3 ± 2.1	1.9 ± 1.2	0.62 (NS)
*P* value (lesion versus perilesional skin)	*P* < 0.003	*P* < 0.05	

NS: not significant.

**Table 2:** Serum level of IL-17-in examined groups.

IL-17			
	Control	DH	BP
IL-17	5.48	28.10	24.05
SEM	3.11	1.36	0.78
SD	8.229	6.375	7.471

Control	NS	*P* < 0.001	*P* < 0.001
DH	*P* < 0.001	NS	*P* < 0.05
BP	*P* < 0.001	*P* < 0.05	NS
